# P-751. Clinical Features, Microbiological Epidemiology, and Recurrence Risk of Cellulitis in Breast Cancer-Related Lymphedema

**DOI:** 10.1093/ofid/ofaf695.962

**Published:** 2026-01-11

**Authors:** Benjamin D Wagner, Jonathan Rubin, roshni Culas, Anna Kaltsas, Maryam Abul, Jilmil Raina, I Hsin Lin, Bracha Pollack, Arielle Roberts, Andrea Barrio, Babak Mehrara

**Affiliations:** Memorial Sloan Kettering Cancer Center, New York, New York; Memorial Sloan Kettering Cancer Center, New York, New York; Memorial Sloan Kettering Cancer Center, New York, New York; Memorial Sloan Kettering Cancer Center, New York, New York; Memorial Sloan Kettering Cancer Center, New York, New York; Memorial Sloan Kettering Cancer Center, New York, New York; Memorial Sloan Kettering Cancer Center, New York, NY, USA, NYC, New York; Memorial Sloan Kettering Cancer Center, New York, New York; Memorial Sloan Kettering Cancer Center, New York, New York; Memorial Sloan Kettering Cancer Center, New York, New York; Memorial Sloan Kettering Cancer Center, New York, New York

## Abstract

**Background:**

Cellulitis is a complication for patients with breast cancer-related lymphedema (BCRL). This study evaluates the prevalence, clinical presentation, bacterial epidemiology, and treatment outcomes of cellulitis in BCRL, with a goal of identifying factors associated with recurrence.

**Figure:**
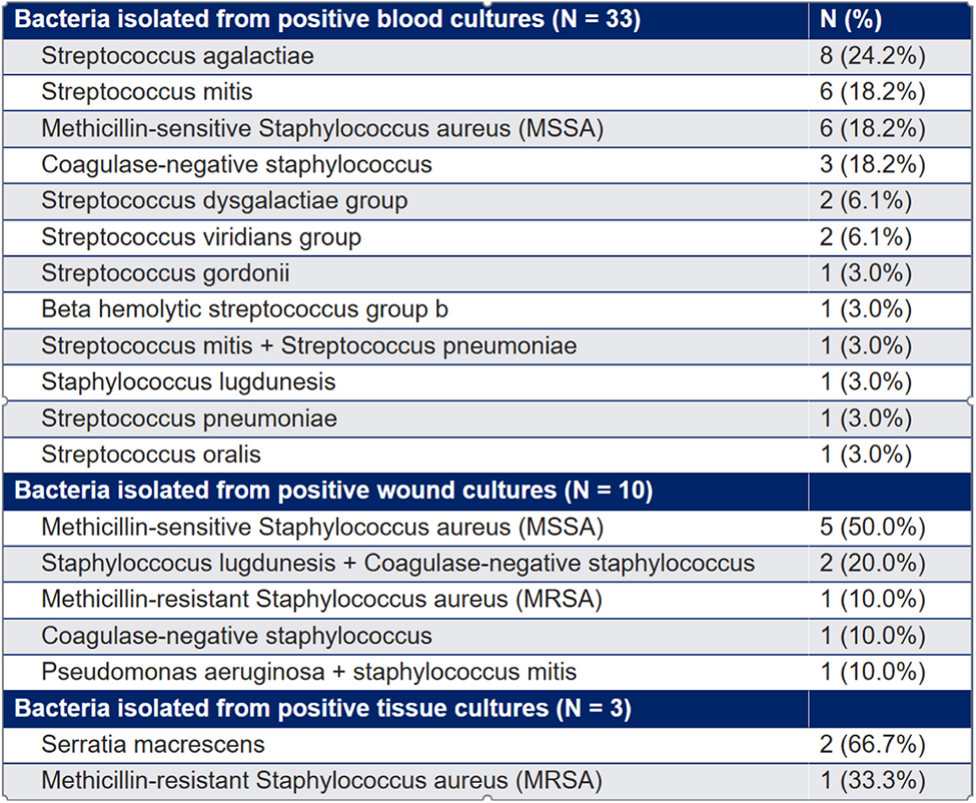
Microbiological Epidemiology of ​ Cellulitis in BCRL Blood cultures were obtained in 61% (255/418) of episodes​, 12.9% (33/255) returned positive​ Streptococcus agalactiae (8/33) was the most common bacteria isolated from positive cultures

**Figure:**
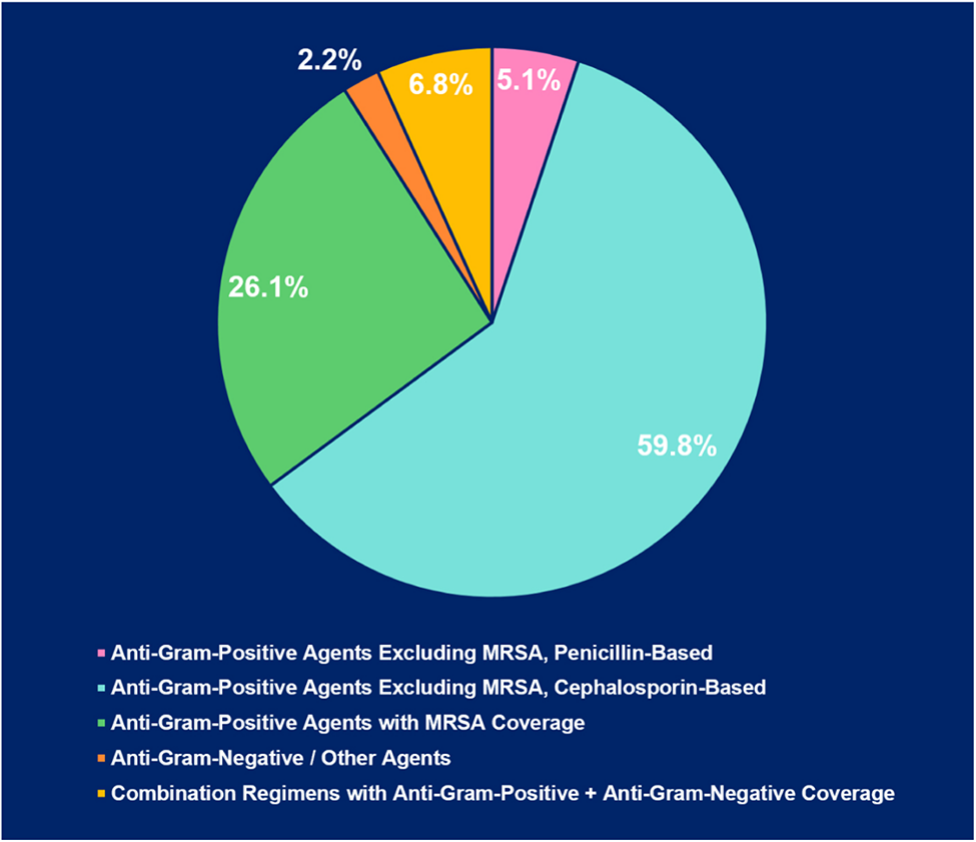
Treatment Overview and Antibiotic Effectiveness in BCRL Cellulitis Effectiveness of Initial Antibiotic Regimen​: 87.6% Effectiveness of Antibiotic Prophylaxis (n 29): 58.6%

**Methods:**

This was a retrospective analysis of cellulitis events between December 2000 -November 2024 in a cohort of 2,920 BCRL patients at Memorial Sloan Kettering Cancer Center. Data on demographics, symptoms, laboratory findings, microbiological cultures, and antibiotic regimens were analyzed. Univariate and multivariable Cox Proportional Hazard models were applied.

**Table 1:**
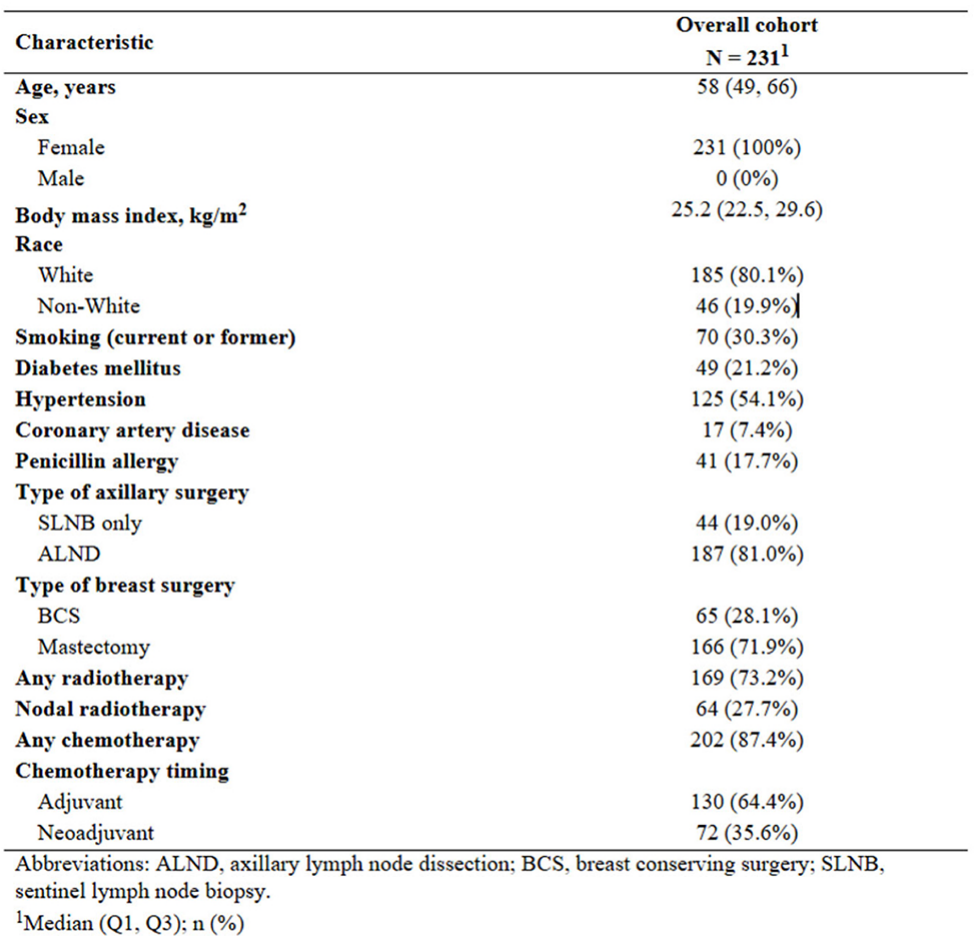
Patient Demographics and Other ​Characteristics (N = 231)

**Table 2:**
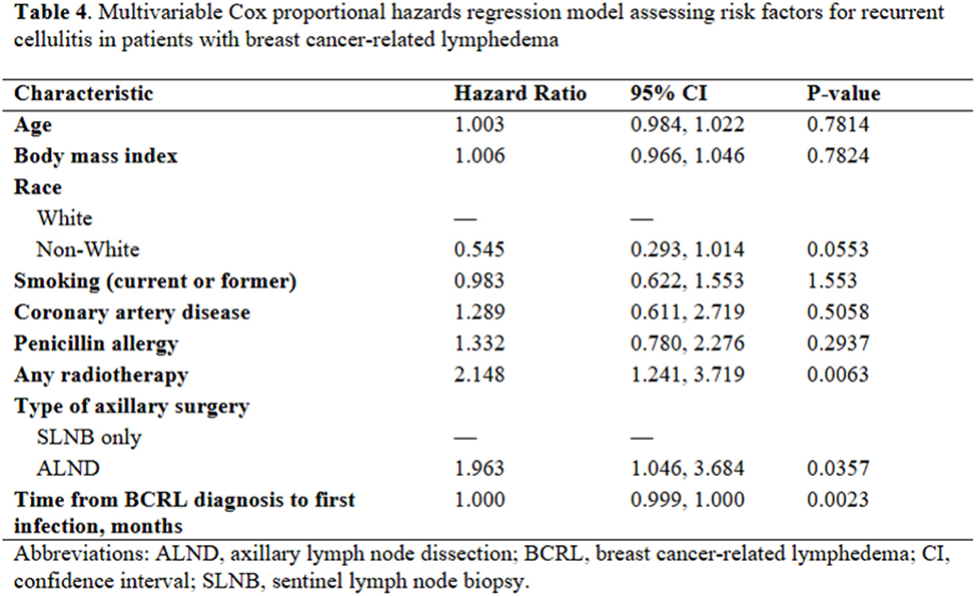
Multivariable Cox proportional hazards regression model assessing risk factors for recurrent cellulitis in patients with breast cancer-related lymphedema

**Results:**

A total of 418 cellulitis episodes were documented among 231 BCRL patients, with a cellulitis prevalence of 7.9% (231/2,920) and a recurrence rate of 39.0% (90/231). The median number of cellulitis episodes per patient was 1 (IQR: 1, 2), with a median follow-up duration of 85.5 months (IQR: 42.8, 137.6). Patients with recurrent cellulitis (≥2 episodes) had a shorter median time from lymphedema onset to first cellulitis episode compared to non-recurrent cases (4.9 months vs. 13.9 months, p=0.014) and were more likely to present with a WBC count >11 K/mcL (29.0% vs. 10.5%; p< 0.001). Blood cultures were obtained in 255 episodes (61.7%), with positive results in 33 cases (12.9%). *Streptococcus agalactiae* was the most common pathogen (8/33; 24.2%). Empiric antibiotic therapy was effective without modification in 87.4% of episodes. The most common first-line antibiotics were cephalosporin-based agents targeting gram-positive bacteria, excluding MRSA (59.5%), followed by agents with MRSA coverage (26.2%). Among those receiving prophylactic antibiotics, 58.6% (17/29) had no recurrent infections. Multivariable analysis showed that radiation, node dissection and time from lymphedema to infection #1 were independently associated with risk of recurrence of infection.

**Conclusion:**

Our findings suggest that radiation, node dissection, and time from lymphedema diagnosis to first cellulitis episode were significantly associated with risk of recurrent cellulitis.

**Disclosures:**

All Authors: No reported disclosures

